# HEALTH PROMOTION BEHAVIORS AMONG SPOUSAL CAREGIVERS WITH HIGH DAILY MANAGEMENT CHRONIC CONDITIONS

**DOI:** 10.1093/geroni/igae098.3409

**Published:** 2024-12-31

**Authors:** Jinmyoung Cho, Molly Horstman, Alan Stevens, Laura Sands, Heather Allore

**Affiliations:** Saint Louis University School of Medicine, Ballwin, Missouri, United States; Baylor College of Medicine, Houston, Texas, United States; Baylor Scott & White Health, Belton, Texas, United States; Virginia Tech, Blacksburg, Virginia, United States; Yale University, New Haven, Connecticut, United States

## Abstract

As the disease trajectory of dementia increases caregiving intensity, family caregivers are less likely to take care of their own health. Although the potential health threats from chronic caregiving distress are known, far less is known about health behaviors among caregivers with chronic conditions while caring for a person with dementia (PWD). This study aims to identify caregivers diagnosed with chronic conditions requiring high daily self-management and examine their health promotion behaviors associated with the intensity of caregiving among spousal caregivers for PWD. Using ICD-10 codes from medical records of 152 spousal caregivers aged 65 and older, six chronic conditions requiring high daily self-management (CCHDSM) were asthma, chronic kidney disease, chronic obstructive pulmonary disease, diabetes, heart failure, and ischemic heart disease. The intensity of caregiving was identified by cluster analysis (high vs. low). Self-rated health promotion behaviors were assessed as: health responsibility, physical activity, nutrition, interpersonal relations, and stress management. Ninety-nine caregivers (61.5%) were diagnosed with at least one CCHDSM. No significant difference in the intensity of caregiving was observed between CCHDSM and non-CCHDSM caregivers. A significant interaction between the intensity of caregiving and CCHDSM in performing physical activities (F=5.03, p<.01) indicates that caregivers with CCHDSM and providing high intensity of caregiving were the least engaged in physical activities compared to others. Findings highlight caregiving role hinders physical activities for spousal caregivers in the most needs of physical activities. Future studies, interventions, and support systems are urgently needed to reduce burden of caregiving for spousal caregivers struggling with chronic conditions.

